# Sevoflurane Ameliorates Schizophrenia in a Mouse Model and Patients: A Pre-Clinical and Clinical Feasibility Study

**DOI:** 10.2174/1570159X20666220310115846

**Published:** 2022-11-15

**Authors:** Tianyun Zhao, Ziwen Shi, Nongxi Ling, Jingwen Qin, Quancai Zhou, Lingzhi Wu, Yuansheng Wang, Chuansong Lin, Daqing Ma, Xingrong Song

**Affiliations:** 1 Department of Anesthesiology, Guangzhou Women and Children’s Medical Center, Guangzhou Medical University, Guangzhou, China;; 2 Department of Psychiatry, The Third People's Hospital of Xinhui District, Guangdong, China;; 3 Division of Anaesthetics, Pain Medicine & Intensive Care, Department of Surgery & Cancer, Faculty of Medicine, Imperial College London, Chelsea and Westminster Hospital, London, UK;; 4 Department of Anesthesiology, Jiangmen Central Hospital, Affiliated Jiangmen Hospital of Sun Yat-sen University, Guangdong, China

**Keywords:** Low-concentration, sevoflurane, E/I balance, social deficits, early response, schizophrenia

## Abstract

**Background:**

GABAergic deficits have been considered to be associated with the pathophysiology of schizophrenia, and hence, GABA receptors subtype A (GABA_A_Rs) modulators, such as commonly used volatile anesthetic sevoflurane, may have therapeutic values for schizophrenia. The present study investigates the therapeutic effectiveness of low-concentration sevoflurane in MK801-induced schizophrenia-like mice and schizophrenia patients.

**Methods:**

Three weeks after MK801 administration (0.5 mg kg^-1^, i.p. twice a day for 5 days), mice were exposed to 1% sevoflurane 1hr/day for 5 days. Behavioral tests, immunohistochemical analysis, western blot assay, and electrophysiology assessments were performed 1-week post-exposure. Ten schizophrenia patients received 1% sevoflurane 5 hrs per day for 6 days and were assessed with the Positive and Negative Syndrome Scale (PANSS) and the 18-item Brief Psychiatric Rating Scale (BPRS-18) at week 1 and week 2.

**Results:**

MK801 induced hypolocomotion and social deficits, downregulated expression of NMDARs subunits and postsynaptic density protein 95 (PSD95), reduced parvalbumin - and GAD67-positive neurons, altered amplitude and frequency of mEPSCs and mIPSCs, and increased the excitation/inhibition ratio. All these changes induced by MK-801 were attenuated by sevoflurane administration. Six and eight patients achieved a response defined as a reduction of at least 30% in the PANSS total score at 1^st^ and 2^nd^ week after treatments. The BPRS-18 total score was found to be significantly decreased by 38% at the 2^nd^ week (p < 0.01).

**Conclusion:**

Low-concentration sevoflurane effectively reversed MK801-induced schizophrenia-like disease in mice and alleviated schizophrenia patients’ symptoms. Our work suggests sevoflurane to be a valuable therapeutic strategy for treating schizophrenia patients.

## 1 INTRODUCTION

Schizophrenia is a chronic and devastating neurodevelopmental disorder, and affects up to 1% population worldwide [1]. It is ranked among the top 25 leading causes of disability globally and inflicts substantial social, healthcare, and economic burdens [2]. Schizophrenia is clinically characterized by both positive (*i.e.*, hallucinations, delusions, abnormal motor behavior) and negative symptoms (*i.e.*, avolition and sociality) as well as cognitive deficits [3]. Antipsychotics are the standard medications for schizophrenia, and indeed, dopamine or/and serotonin modulating antipsychotics remain the primary approved treatments for schizophrenia [4]. However, the majority of these antipsychotics are mainly effective for positive symptoms and leave most patients with negative symptoms and their cognitive dysfunction relatively untreated; thus, patients have a poor lifelong quality of lives [5, 6]. In addition, due to their different affinity towards various synaptic receptors, these drugs are associated with nonadherence owing to their side effects, including extrapyramidal, cardiotoxicity and abnormal metabolic syndrome (hyperglycemia, hyperlipidemia, weight gain), making them not ideal for long term use [7, 8]. The acute phase, such as the first episode or an acute exacerbation of schizophrenia, is a pivotal phase because optimal treatment for the episode might improve the long-term prognosis [9]. Early diagnosis and prompt and non-coercive interventions to relieve the symptoms during the acute episode are critical to prevent aggressive behavior and symptomatic escalation. Indeed, delayed treatments and poor treatment compliance are considered to be significant barriers for the effective management of psychiatric conditions and the establishment of therapeutic alliance between patients and healthcare providers. Therefore, it is urgently needed to develop therapies with rapid onset of action, which are easy to administer, non-invasive, have good safety profile and favorable tolerability.

γ-aminobutyric acid type A receptors (GABA_A_Rs) are a family of ligand-gated ion channels essential for the regulation of synaptic inhibition and the maintenance of excitation/inhibition (E/I) balance required for normal brain function [10, 11]. Aberrant GABA_A_R activities or dysregulation thereof are associated with various psychiatric diseases, including schizophrenia [12]. With growing evidences implicating GABAergic deficits in schizophrenia, such as reduced parvalbumin (PV) immunoreactive GABA interneurons, diminished expression of glutamic acid decarboxylase67 (GAD67) accounting for most GABA synthesis, and altered types of GABA_A_R subunits and GABA genes in the cortex, targeting GABA_A_R accessory proteins and molecules modulating GABAergic system signaling, might shed light on th need for the development of novel therapeutics for psychotic disorders [11]. In addition, susceptible genes, such as NRG1 (Neuregulin 1), and its receptor ErbB4, have received considerable attention as a plausible pathological mechanism of schizophrenia due to their crucial role in neurodevelopment and modulation of glutamatergic and GABAergic signaling [13].

Sevoflurane, a commonly used volatile anesthetic for surgical anesthesia, is a potentiator of GABA_A_Rs and is increasingly used as a sedative in outpatient departments and intensive care units due to its rapid onset and well-controlled action [14-16]. The present study, therefore, aims to test our hypothesis that inhaling low-concentration of sevoflurane attenuates the abnormal behaviours, and reverses the changes to GABA_A_R associated proteins and related neuregulin-1 (NRG1) and its tyrosine kinase receptor ErbB4 signaling in a mice model of dizocilpine (MK801)-induced schizophrenia [13]. We also evaluated the therapeutic benefit of sevoflurane at low concentration in combination with α_2_-adrenergic agonist dexmedetomidine in a preliminary study comprising a small group of schizophrenia patients. Dexmedetomidine was used to primarily enhance study compliance and prevent the incidence of nausea and vomiting [14].

## 2 MATERIALS AND METHODS

### 2.1 Pre-Clinical Studies

#### 2.1.1 Subjects

All experimental procedures were performed in accordance with the guidelines of the Animal Experiment Ethics Committees of Guangzhou Medical University, Guangzhou, China, and the ARRIVE. Timed-pregnant Balb/c mice were purchased from Guangdong Medical Laboratory Animal Center, Guangzhou, China. On the P7, male pups weighing 4-6 g were randomly allocated to the control group (CTRL), MK801 group (MK801), and MK801+Sevoflurane (MK801+SEV) group. At the time of weaning (P21), mice were separated from their mothers and housed in groups of four to six per cage. MK801 (M107, St. Louis, MO, USA) was used to induce negative symptoms of schizophrenia, as reported previously [17]. In brief, three weeks after MK801 administration (0.5 mg kg^-1^, i.p. twice a day for 5 days), mice were exposed to 1% sevoflurane *via* 1 L min^-1^ in 30% oxygen balanced with nitrogen 1hr/day for 5 days. Detailed methods are provided in the Supplemental file **S1**.

#### 2.1.2 Behavioural Tests and Laboratory Detection

Behavioural tests consist of an open field test (OFT, to assess spontaneous locomotor activity) and a three-chamber sociability test. Open-field test was performed at P43-P45, followed by a three-chamber social test after two non-stimuli days at P47. After behavioural tests, subjects were sacrificed under terminal anaesthesia, and brain samples were harvested for electrophysiological recordings, western blot and immunohistochemical analyses, respectively. The experimental timeline is presented in Fig. (**1a**), and the detailed methods are described in Supplementary File **S1**.

### 2.2 Open-labeled Single-Arm Trial

An open-labeled single-arm, proof-of-concept clinical trial (registered in Chinese Clinical trial; ChiCTR 1900024677) was conducted from September 24, 2019, to January 20, 2020, in the Third People's Hospital of Xinhui District, Guangdong. After obtaining approval from the Ethics committee of Guangzhou Women and Children’s Hospital, Guangzhou, and the Third People's Hospital of Xinhui District, Guangdong, China, and a written informed consent from patients or their family members, ten schizophrenia patients (7 men and 3 women; age 18-65 years) who were taking antipsychotics, experiencing an acute exacerbation of psychosis and meeting all the inclusion criteria (Table **S1**), were recruited (Supplementary File **S2**). Sevoflurane at 1% (adjusted between 0.5-1.2% when necessary) was delivered at 2.5 L min^-1^ in oxygen-enriched air (50% oxygen and 50% air of volume ratio) for 5 hrs using a face mask under spontaneously respiration to maintain the deep sedation. This treatment was repeated six times with intervals of 1-2 days for a total of two weeks. The primary endpoint was the percentage of early response at week 2, with clinical response defined as a minimum 30% reduction in the Positive and Negative Syndrome Scale (PANSS) total score. The percent change of PANSS total score was defined as a change /(baseline - 30) x100% [18]. The secondary endpoints were the change of Brief Psychiatric Rating Scale (BPRS-18) from the baseline to week 1 and week 2, and the early response rate at week 1. Safety and tolerability assessments included treatment-emergent adverse events (TEAEs), clinical laboratory tests, electrocardiograms, and physiologic measures. Detailed trial protocol and procedures are provided in the Supplementary File **S3**.

### 2.3 Statistical Analysis

Pre-clinical study: All data have been presented as a dot plot and mean ± SD, and statistical analyses were performed with SPSS (SPSS Inc., Chicago, IL, USA). Treatment effects were statistically analysed by one-way ANOVA followed by Bonferroni’s post-hoc tests for comparison when normality (and homogeneity of variance) assumptions were satisfied; otherwise, Kruskal-Wallis test was applied to analyze the differences between groups followed by Dunn’s multiple comparisons tests. Bodyweight was analysed using a two-way ANOVA followed by Bonferroni’s post-hoc tests. A p-value < 0.05 was considered to be of statistical significance.

Clinical study: Simon’s two stage design was used in this proof of concept study. Based on findings of an earlier study, the percentage of patients with at least 30% improvement in total PANSS at 2 weeks was about 30% [19]. In this study, a 70% or higher early response rate was set as the target of “good” and 30% was set as “poor”. The optimal two-stage design was used to test the null hypothesis that P<=0.300 versus the alternative hypothesis that P>=0.700 has an expected sample size of 6.08. After testing the drug on 2 patients in the first stage, the trial was planned to be terminated if 0 responded. If the trial went on to the second stage, a total of 10 patients were planned to be studied. If the total number of patients responding was less than or equal to 5, the drug was rejected. The efficacy and safety analyses were conducted in the full analysis set, which included all patients who received at least one treatment of sevoflurane inhalation and had at least one evaluation post-baseline. Data were expressed as mean ± standard deviation or median with range and were then presented as a scatter plot or Box-Whisker plot, wherever appropriate.

## 3 RESULTS

### 3.1 Pre-Clinical Data

#### 3.1.1 MK801 Caused a Persistent Bodyweight Reduction in Mice

We measured body weight on a daily basis during MK801 treatment and once a week thereafter till the P32. Two-way ANONA with repeated measures revealed persistent lower body weight from P11 to P31 in MK801-treated mice compared to controls (Fig. **S1**), (n = 18, *P* < 0.01 at P11, and *P* < 0.01 at the P17, P24 and P31).

#### 3.1.2 Sevoflurane Treatment Rescued Mk801-induced Hypoactivity in the OFT

**Fig. (1):**
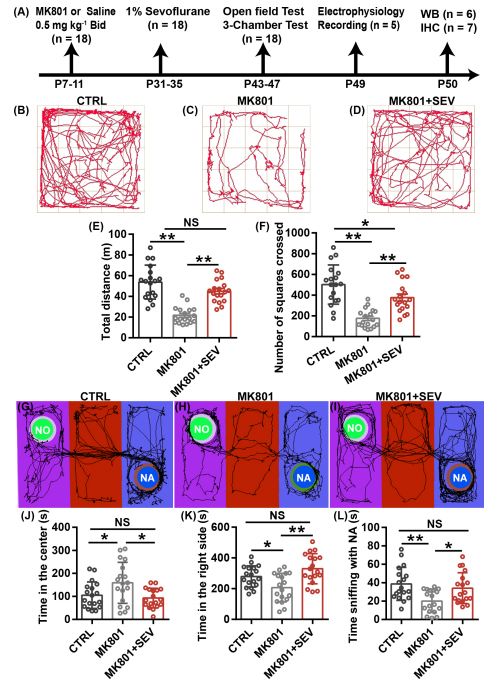
**Sevoflurane (1%) for 1hr for five consecutive days protected against MK801-induced hypoactivity in the open field test (OFT) and social interaction defects.** (**A**) P7 mice received intraperitoneal (i.p.) injection of 0.5 mg kg-1 MK801 (MK801 group and MK801+SEV group) or an equal volume of saline (CTRL group) twice a day for five consecutive days. During the P31-P35, mice were exposed to 1% sevoflurane *via* 1 L min-1 in 30% oxygen balanced with nitrogen 1hr/day for 5 days. Behavioural tests consisted of open field test performed at P43-P45 and a three-chamber sociability test performed at P47. Then mice were sacrificed and brain samples were harvested for electrophysiological recordings at P49, western blot and immunohistochemical staining at P50, respectively. (B to D) The recording trail traces from the CTRL (**B**), MK801 (**C**) and MK801+SEV (**D**) group, respectively. (**E**) Statistics showed a significant difference in total distance travelled in the OFT among the three groups. (F) Statistics showed a significant difference in the frequency of squares crossed in the OFT among the three groups. (**G** to **I**) Examples of recording trails from CTRL (**G**), MK801 (**H**), and MK801+SEV group (**I**), respectively. (**J**) Statistics showed a significant difference in time spent in the middle chamber among the three groups. (**K**) Statistics showed a significant difference of time spent in the right chamber among the three groups. (**L**) Statistics showed a significant difference of time sniffing NA (naive animal). One-way ANOVA, followed by Bonferroni’s *post-hoc* tests, was used for analysis. Data are represented as mean ± SD (n = 18); *P < 0.05; **P < 0.01. CTRL: control group; SEV: Sevoflurane.

OFT was performed 7 days after sevoflurane exposure to investigate the spontaneous locomotor activity; the trial examples in each group are shown in Fig. (**1B**-**D**). Sevoflurane exposure (MK801+SEV) significantly increased the total distance compared to that in the MK801 group, while exhibiting no significant difference compared to the CTRL group (Fig. **1E**), (n = 18, *P* < 0.001). Similarly, MK801+SEV mice displayed significantly higher frequency of crossing squares compared to the MK801 group while having no difference compared to the CTRL group (Fig. **1F**), (*P* < 0.001).

#### 3.1.3 Sevoflurane Treatment Restored MK801-induced Social Interaction Defects

During the social approach phase, the test mice (n = 18) in three groups explored freely in each chamber, as shown in the examples of recording trials (Fig. **1G** to **I**, CTRL: left; MK801: middle; MK801+SEV: right) and density maps (Fig. **S2**). There was no difference observed in the time for exploration of the left chamber with an empty black wire cup inside (data was not shown), while the times for exploring the center chamber (Fig. **1J** and F = 3.848, *P* < 0.05) and the right chamber with a novel mouse inside the wire cup (Fig. **1K** and F = 6.982, *P* < 0.01) were significantly different. Bonferroni’s *post-hoc* tests showed that MK801-treated mice spent more time lingering in the center chamber and less time in the right chamber than mice in the CTRL and MK801+SEV groups (Fig. **1J** and K, *P* < 0.05 and *P* < 0.01, respectively). Sevoflurane exposure (MK801+SEV) increased the time for sniffing of the novel animal than the MK801 group mice (Fig. **1L**, *P* < 0.05); there was no significant difference observed between the MK801+SEV and CTRL groups (Fig. **1L**, *P* = 0.659).

#### 3.1.4 Sevoflurane Reversed MK801-induced Aberrant NMDARs Composition in the mPFC

**Fig. (2):**
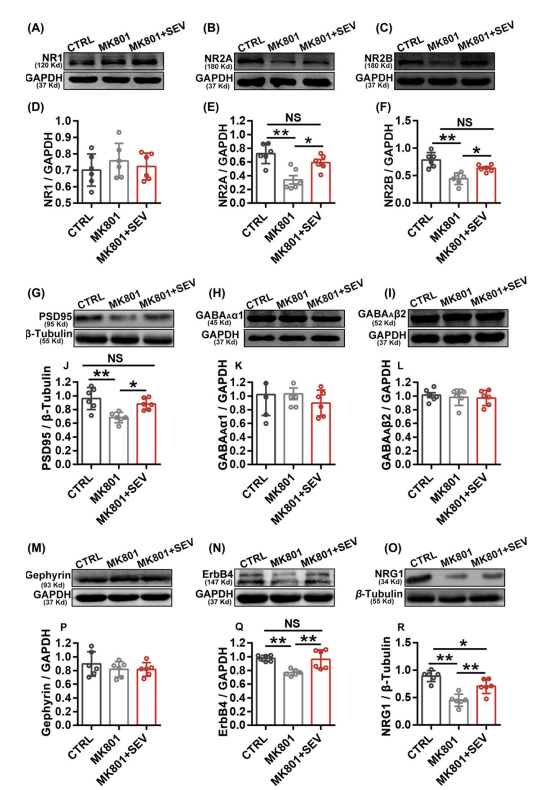
**Sevoflurane (1%) for 1hr for consecutive 5 days reversed MK801-induced aberrations to NMDARs composition and ErbB4-NRG1 signal pathway in the mPFC.** (**A** to **C**) Representative bands of NMDAR subunit NR1, NR2A and NR2B in the mPFC, respectively. (**D** to **F**) Quantification graphs comparing the band intensities of NR1, NR2A and NR2B between three groups. (**G** to **I**) Representative bands of PSD95, GABAAR subunit α1 and GABAAR subunit β2 in the mPFC, respectively. (**J** to **L**) Quantification graphs comparing the band intensities of PSD95, GABA_A_R subunit α1 and GABA_A_R subunit β2 between the three groups. (**M** to **O**) Representative bands of the GABA_A_R anchoring protein gephyrin, ErbB4 and NRG1 in the mPFC, respectively. (**P** to **R**) Quantification of the band intensities of gephyrin, ErbB4 and NRG1 between the three groups. One-way ANOVA, followed by Bonferroni’s *post-hoc* tests, was used for analysis. Data are represented as mean ± SD (n = 6 per group); *P < 0.05; **P < 0.01. CTRL: control group; SEV: Sevoflurane.

The expression of N-methyl-D-aspartate receptors (NMDARs) subunits, including NR1, NR2A, NR2B, and the scaffold protein PSD95 within the medial prefrontal cortex (mPFC) were determined by Western blot assay. There were no significant differences found in the expression of NR1 in the three groups (Fig. **2A** and **D**, n = 6, *P* = 0.602). However, the expressions of NR2A, NR2B and PSD95 were significantly decreased in the MK801 group compared to CTRL (Fig. **2B**-**J**, *P* < 0.01), and reversed in the MK801+SEV group (Fig. **2E**, **F** and **J**, *P* > 0.05).

#### 3.1.5 Sevoflurane Reversed MK801-induced Alterations of GABAergic Neuronal Transmission and ErBb4-NRG1 Signal Pathway in the mPFC

No differences in the expression of GABA_A_ receptor subunits were observed, including α1, *β*2, and the anchoring protein gephyrin (Fig. **2H** and **K**, GABA_A_ α1, n = 6, *P* = 0.568; I and L, GABA_A_ *β*2, *P* = 0.784; M and P, Gephyrin, *P* = 0.507, respectively). Furthermore, the ErbB4-NRG1 signal pathway was explored to see whether it is related to the underlying mechanism that sevoflurane acts through to ameliorate schizophrenia. ErbB4 level was significantly suppressed in the MK801 group when compared to CTRL (Fig. **2N** and **Q**, *P* < 0.01), while MK801+SEV reversed such decrease (Fig. **2Q**, *P* < 0.01). Likewise, MK801 significantly upregulated NRG1 expression compared to CTRL (Fig. **2O** and R, *P* < 0.001), and sevoflurane prevented MK801-induced NRG1 expression (Fig. **2**, R, *P* < 0.01).

**Fig. (3):**
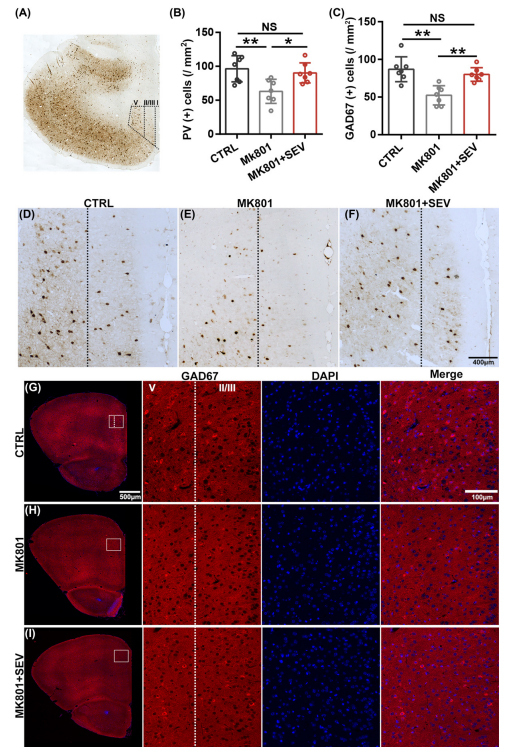
**Sevoflurane (1%) for 1hr for consecutive 5 days rescued MK801-induced reductions of PV interneurons and GAD67 positive interneurons in the mPFC.** (**A**) Schematic illustration indicates the mPFC (dotted box) where images were taken for analysis. (**B**, **C**) Statistic analysis showed significant decreases of PV interneurons (**B**) and GAD67 positive interneurons (**D**) in the MK801 group compared to the CTRL group, which were re-elevated in the MK801+SEV group. (**D** to **F**) Representative images of PV interneurons in the CTRL (**D**), MK801 (**E**) and MK801+SEV (**F**) groups, respectively. Scale bars were 400 μm in **D** to **F**. (**G** to **I**). The left column represents anti-GAD67 immunofluorescence staining of the CTRL (**G**), MK801 (**H**) and MK801+SEV (**I**), with a boxed area showing high-resolution images taken for analysis. The black dotted lines in A, and D to F and the white dotted lines in G, H, and I indicate the borders of different laminae in the mPFC. Scale bars were 500 μm in the first panel and 100 μm in the rest of the panels. One-way ANOVA followed by Bonferroni’s *post-hoc* tests was used for all analyses. Data are represented as mean ± SD (n = 7); *P < 0.05; **P < 0.01. CTRL: control group; SEV: Sevoflurane.

We also detected a significant difference in the number of parvalbumin (PV) positive interneurons in the mPFC, as indicated by the boxed area corresponding to the prelimbic area (Fig. **3A**, **D** to **F**, n = 7, *P* < 0.01). Specifically, the density of PV interneurons was significantly decreased in the MK801 group compared to that of the CTRL (Fig. **3**, **B**, *P* < 0.01), while the density of PV interneurons in the MK801+SEV group was increased compared to MK801 alone (Fig. **3B**, *P* < 0.05). The density of glutamic acid decarboxylase 67 (GAD67) positive cells was also different amongst the three groups (Fig. **3C**, n = 7, *P* < 0.001; Fig. **3G** to **I**), with MK801 significantly reducing the density of GAD67 positive cells compared to that of CTRL, which was reversed in MK801+SEV group (Fig. **3C**, *P* < 0.01).

#### 3.1.6 Sevoflurane Reversed MK801-induced Alterations of Electrophysiological Profiles and E/I Balance in the mPFC

**Fig. (4):**
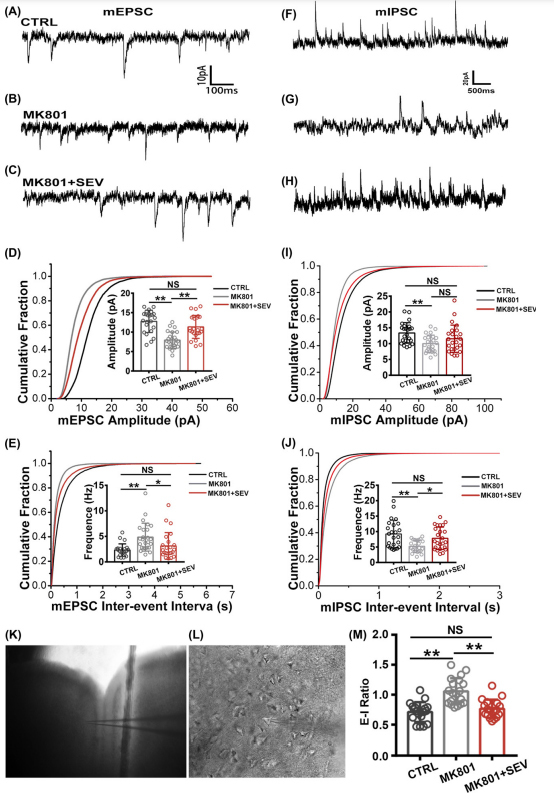
**Sevoflurane (1%) for 1hr for consecutive 5 days reversed MK801-induced alterations to electrophysiological profiles and E/I balance in the mPFC.** (A to C) Representative mEPSC recording traces of pyramidal neurons in laminae II/III of the mPFC from the CTRL (**A**), MK801 (**B**) and MK801+SEV (**C**) mice. (**D**) Cumulative distribution of mEPSC amplitudes recorded from the CTRL (black), MK801 (gray) and MK801+SEV (red) mice. Inset, mean amplitude of mEPSCs of the CTRL (23 cells), MK801 (25 cells) and MK801+SEV (24 cells) group mice. (**E**) Cumulative distribution of mEPSC inter-event intervals recorded from the CTRL (black), MK801 (gray) and MK801+SEV (red) group mice. Inset, mean frequency of mEPSCs of the CTRL (23 cells), MK801 (25 cells) and MK801+SEV (24 cells) mice. (**F** to **H**) Representative mIPSC recording traces of pyramidal neurons in laminae II/III of the mPFC from the CTRL (F), MK801 (G) and MK801+SEV (**H**) mice. (**I**) Cumulative distribution of mIPSC amplitudes recorded from the CTRL (black), Mk801 (gray) and MK801+SEV (red) mice. Inset, mean amplitude of mIPSCs of the CTRL (24 cells), MK801 (24 cells) and MK801+SEV (24 cells) group mice. (**J**) Cumulative distribution of mIPSC inter-event intervals recorded from CTRL (black), MK801 (gray) and MK801+SEV (red) group mice. Inset, mean frequency of mIPSCs of the CTRL (24 cells), MK801(24 cells) and MK801+SEV (24 cells) mice. (K and L) Laminar II/III of the mPFC pyramidal neurons viewed under infrared optics was recorded with a patch-clamp recording pipette. (M) The E/I ratio showed a significant increase in MK801 mice (27 cells) compared to the CTRL mice (26 cells), while the E/I ratio in MK801+SEV (28 cells) mice was significantly decreased compared to MK801 mice. One-way ANOVA followed by Bonferroni’s *post-hoc* tests and Kruskal-Wallis tests followed by Dunn’s multiple comparison tests were used for analysis. Data are represented as mean ± SD (5 mice per group); *P < 0.05; ** *P* < 0.01. CTRL: control group; SEV: Sevoflurane.

To confirm whether sevoflurane treatment can reverse MK801-induced alterations to electrophysiological characteristics of pyramidal neurons in the laminar II/III of the mPFC, whole-cell recordings were performed 2 weeks after sevoflurane treatment at P49 (n = 5). The excitatory postsynaptic currents (mEPSCs) recorded (Fig. **4A**-**C**) in the mPFC pyramidal neurons of the CTRL, MK801 and MK801+SEV demonstrated that MK801 significantly suppressed the mean amplitude compared to CTRL (Fig. **4D**, *P* < 0.001), which was re-elevated in the MK801+Sevo group (Fig. **4D**, *P* < 0.001). Unexpectedly, in the MK801 group, the frequency cumulative distribution plot showed a distinct leftward shift, with a significant increase in the mean frequency of miniature excitatory postsynaptic currents (mEPSCs) compared to the CTRL (Fig. **4E**, *P* < 0.001), while the mean frequency of mEPSCs was reversed in the MK801+SEV group, as compared to the MK801 group (Fig. **4E**, *P* < 0.05). In addition, the analysis of the miniature inhibitory postsynaptic currents (mIPSCs) recorded (Fig. **4F**-**H**) in the mPFC pyramidal neurons indicated that MK801 decreased the frequency and amplitude of mIPSCs relative to the controls (Fig. **4I** and **J**, *P* < 0.001), while MK801 superposed with sevoflurane significantly reversed the reduction of frequency but not the amplitude of mIPSCs (Fig. **4I**). Furthermore, pyramidal neurons of laminar II/III of the mPFC (Fig. **4K**) were viewed under infrared optics and recorded for mEPSCs and mIPSCs in the same neuron (Fig. **4L**). The E/I ratio (mEPSC/mIPSC amplitude of the same pyramidal neuron) was significantly different between the groups (Fig. **4M** F = 19.630, *P* < 0.001), whereby E/I ratio in the MK801 group was higher than in the CTRL group (Fig. **4M**, *P* < 0.001), which suggested a tendency towards hyperexcitation; MK801+SEV group significantly decreased E/I ratio compared to that of MK801 (Fig. **4M**, *P* < 0.001).

### 3.2 Clinical Data

**Fig. (5):**
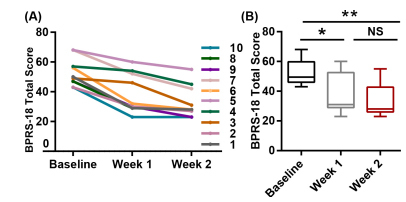
**Low-concentration sevoflurane improved schizophrenia patients’ symptoms.** (**A**) Individual 18-item Brief Psychiatric Rating Scale (BPRS-18) total scores were assessed at week 1 and week 2 after initiation of sevoflurane inhalation. (**B**) Statistics comparing BPRS-18 scores at week 1 and week 2 to baseline score (n = 10, One-way ANOVA followed by Tukey’s *post-hoc* analysis; *P < 0.05, ** *P* < 0.01). CTRL: control group; SEV: Sevoflurane.

Nine out of ten patients enrolled (seven male and three female) completed all 6 trial treatments, and because of postural hypotension after treatment, 1 patient withdrew after receiving one treatment (a male patient-No.3 in Fig. **5A**). Data from all patients were included in the final data analysis. The clinical and demographic characteristics of the patients and their baseline PANSS and BPRS scores are shown in Supplementary Table **S2**.

#### 3.2.1 Primary Efficacy

Eight patients achieved 30% decrease in PANSS total score from baseline to the end of week 2 following sevoflurane treatment (Table **S3**). A total of six patients out of 10 patients (full analysis set) achieved a 30% decrease in PANSS total score from baseline to the end of week 1 following sevoflurane treatment. The BPRS-18 total score revealed a rapid decrease from the baseline to week 1 and week 2. The BPRS-18 total score significantly decreased over time with a mean (±SD) change from a baseline of -14.30 (±7.39) at the end of week 1 to -19.80 (±5.31) at the end of week 2 (Fig. **5B**). One patient with a disease history of 16 years received one electroconvulsive therapy, given that the clinical symptoms did not improve after three treatments.

#### 3.2.2 Safety

No abnormal physiological parameters and no sevoflurane-related side effects were found, and patients tolerated the treatment well. Liver and kidney functions were not affected by the treatment when compared to the baseline (Fig. **S3**).

## 4 DISCUSSION

The present study aimed to investigate the potential therapeutic effects of low-concentration sevoflurane inhalation on behavior deficits and perturbations to glutamatergic and GABAergic neurotransmission in an MK801-induced mice model that mimics negative features of schizophrenia. Our findings were as follows: i) Mice treated with MK801 at 0.5 mg kg^-1^ (twice a day for 5 days) displayed hypolocomotion and impaired sociability, and had a lower body weight due to its systemic toxicity *per se*; ii) Low-concentration of sevoflurane reversed these behavioral deficits; iii) Low-concentration of sevoflurane attenuated neurochemical and electrophysiological alterations, and restored NMDA glutamatergic and GABAergic neurotransmission. Our findings indicate that sevoflurane may be a potential therapy for treating an acute episode and negative symptoms of psychotic disorders.

Previous studies suggest the glutamatergic and GABAergic neurons and their neurotransmitter systems to play crucial roles in the pathophysiology of schizophrenia [11]; but, currently, there are no relevant new medications available for clinical use. Nevertheless, we found that 1 hour of 1% sevoflurane inhalation for 5 days reversed the decreases in locomotion and sociability induced by MK801. OFT was adopted to assess the exploratory behaviors and emotional disorders, including anxiety-like and depression-like behaviors [20, 21], whilst an automated three-chambered social approach task was used for evaluating sociability [22]. Our results agree with studies showing that sub-chronic administration of MK801 to rodents induced a reduction in spontaneous locomotor activity in the long-term, which may reflect psychomotor retardation [23, 24].

Glutamate (Glu) and GABA, as the major excitatory and inhibitory neurotransmitters in the brain, respectively, are crucial for maintaining normal brain function. NMDARs, which predominantly localize at the excitatory postsynaptic membrane and interact with the postsynaptic density 95 (PSD95), are ionotropic glutamate receptors comprised of two conserved NR1 subunits and two NR2 and/or NR3 subunits. The heteromeric NMDARs provide a wide variety of well-organized compositions of subunits to engage in specific neuronal functions within different brain regions. Therefore, it is not surprising that disturbed composition, misplacement and abnormal trafficking of NMDAR subunits are found in pathological conditions, psychiatric diseases and developmental disorders [25]. NMDARs show rapid maturation by switching from NR2B- to NR2A-NMDARs during the early development in mice and are prone to MK801-induced schizophrenia-like symptoms [23, 26, 27], And NMDAR antagonists, *e.g.,* phencyclidine (PCP) and MK801, can induce symptoms of schizophrenia in normal human subjects [28]. Therefore, different paradigms and regimens of non-competitive NMDAR antagonists, such as ketamine, PCP or MK801, have been extensively used in animal pharmacological models of schizophrenia research to mimic certain aspects of the negative symptoms, such as reduced locomotor activity, social interaction deficits and cognitive impairments [17]. In our study, sub-chronic administration of MK801 during the rapid neurodevelopmental phase led to the disturbed composition of NMDARs subunits, with downregulation of NR2A and NR2B subunits and the scaffolding protein PSD95, but NR1 subunit expression remained unchanged. These pattern changes of NMDARs subunits were also found in the cortex of postmortem brain of patients with schizophrenia, as reported previously [29, 30]. NRG1, NR2 subunits and ErbB4 share a common anchoring region on the postsynaptic sites, and NRG1- ErbB4 signaling can affect NMDAR and its function [31]. Indeed, we found the expressions of NRG1, ErbB4, NR2A, NR2B and PSD95 to be significantly decreased following MK801 administration and low-concentration sevoflurane reversed all these changes induced by MK801. GABA_A_Rs are heteropentameric structures assembled from a large family of subunits, including two α (α1-6), two β (β1-3), and either one γ (γ1-3) or one δ subunit [10]. The majority of GABA_A_Rs comprise α1, β2, and γ2 subunits located at GABAergic synapses *via* direct association with key scaffolding protein gephyrin, which is essential for phasic GABA_A_R signaling [32]. GABA_A_Rs containing α1 and β2 subunits are critical for the sedative action of general anesthetics, such as sevoflurane [33]. Several subunits of GABA_A_R, including α1, α2, β2, β3 and γ2 (the example data of α1 and β2 shown in Fig. **2H** and **I**), have been studied in our study, but no significant changes induced by MK801 or sevoflurane treatment were found. However, GAD67 and PV positive GABA neurons were reduced by MK801 and these reductions were reversed by sevoflurane. Interestingly, how MK801 decreased GABA neurons or their transmitters but did not change GABA receptor subunits is an open question and warrants further study. It is plausible that the α1–3β2/3γ2 of GABA_A_Rs at synapse [34] may not be sensitive to sevoflurane, further raising the question as whether the therapeutic effects of sevoflurane in our study are due to specific targeting of GABA_A_R subunits; this will need to be further investigated. In addition, volatile anesthetics also act on glutamate and acetylcholine neurotransmitter receptors, and potassium and sodium channels [16, 35]. Whether these receptors or channels are involved in the therapeutic effects of sevoflurane warrants further study in the future.

Prefrontal cortex (PFC) plays a fundamental role in regulating multiple complex behaviors, including memory, cognitive, attention, social interaction and emotional regulation. The PV interneurons, the most common subtype of GABAergic neurons known for their fast-spiking phenotype, have strong control over the excitability of pyramidal neurons by innervating the soma and proximal dendrites [36]. Therefore, PV interneurons are powerful regulators for maintaining optimal E/I balance within prefrontal local circuits for information processing. Previously, it has been demonstrated that disrupting E/I balance in the PFC *via* pharmacological, chemogenetic and optogenetic approaches induced a range of PFC-dependent abnormalities associated with psychiatric diseases [12]. MK801 administration likely disrupts the E/I balance in pyramidal neurons in laminar II-III of the mPFC *via* the reduced GABAergic neurotransmission and hypofunctional NMDARs. Indeed, both GAD67 positive cells and PV interneurons were decreased, and this was in line with a previous study showing that the hypofunction of NMDAR led to defective GABA transmission [37, 38]. Furthermore, both excitatory and inhibitory synaptic transmissions in the pyramidal neurons were impaired by MK801 administration, evidenced by the decreased amplitude of mEPSCs and mIPSCs, the increased frequency of mEPSCs, and as a result, increased E/I ratio. MK801 blocks active NMDARs and might preferentially act on NMDARs *via* PV interneurons [39]. Consequently, defective interneuron function from MK801 administration resulted in presynaptic disinhibition of excitatory pyramidal cells, seen as increased frequency distribution in our study; while the decreased amplitude of mEPSCs might be attributed to the hypofunction of postsynaptic NMDARs on pyramidal cells to compensate for increased frequency *per se*. Interestingly, consistent with the increased PV interneurons and GAD67-positive cells contributing to the presynaptic mechanisms of inhibitory synapse and GABA release, a low concentration of sevoflurane also increased GABAergic neurotransmission and reversed these electrophysiological changes induced by MK801 as demonstrated by the increased mean frequency of mIPSCs in the present study. Moreover, it is well known that inhalational anaesthetics can module neural activity [40] and promote hippocampal neurogenesis [41]; all these mechanisms may contribute to the effects of sevoflurane found in the current study, but such possibilities need to be investigated in future studies using mice receiving sevoflurane only without MK801.

Our proof of concept study indicated that patients tolerated the treatment very well, and that symptoms noticeably improved by the treatment. Most importantly, of these patients, five patients with a disease history over 10 years, and whose symptoms that failed to respond to routine anti-schizophrenia medications, improved with this novel therapy. The most likely antipsychotic action of sevoflurane may be attributable to its ability to enhance GABAergic neurotransmission, the deficit of which underlies the pathophysiology of schizophrenia. Other mechanisms may also be involved; for example, the subanesthetic concentration of sevoflurane may increase regional cerebral blood flow [42], which improves brain metabolism and function. Dexmedetomidine, α_2A_-adrenoceptor agonist, was primarily used to enable a smooth induction of sevoflurane sedation, and was maintained throughout the sedative procedure to reduce potential side effects, *e.g.*, postoperative nausea and vomiting, of sevoflurane [43]. Interestingly, it was reported that activation of postsynaptic α_2A_-adrenoceptors in the PFC improves cognitive functions and, therefore, α_2A_-adrenoceptor (α2A-AR) agonists may be promising targets in the antipsychotic therapy [44]. Furthermore, the imbalance between oxidant (such as nitric oxide) and antioxidant systems (*e.g.*, glutathione) is implicated in the underlying pathophysiology of schizophrenia [45-47]. Current published data suggest that both sevoflurane and dexmedetomidine modulate oxidative stress through multiple mechanisms, including nitric oxide production and the glutamate–nitric oxide–cyclic guanosine monophosphate pathway [48, 49]. Nevertheless, to date, neither dexmedetomidine nor sevoflurane is familiar to mental health providers, with a lack of evidence supporting the potential therapeutic values of sevoflurane and/or dexmedetomidine in the treatment of schizophrenia [50, 51]. To this end, whether the beneficial effects we found in our patients were due to the interaction between sevoflurane and dexmedetomidine, or either agent alone, remains a key question that needs further investigation. In addition, acute nephrotoxicity and hepatotoxicity associated with older inhalational anaesthetics, such as methoxyflurane and halothane, were not observed with repeated exposure of sevoflurane in the small pilot study reported here; such potential side effects need to be carefully monitored in larger sample size (ChiCTR1900027767).

## CONCLUSION

**Fig. (6):**
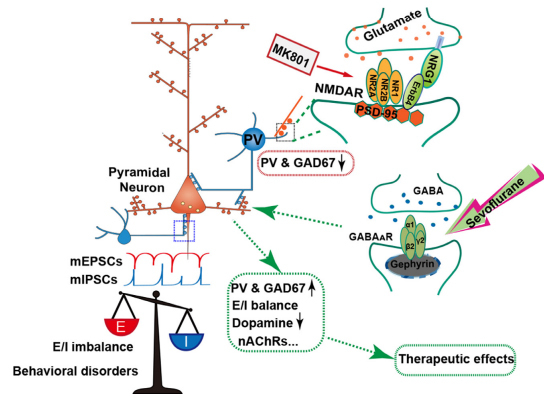
**Schematic illustration of the rectifying imbalance of GABAergic and glutamatergic neurotransmission under sevoflurane exposure in an MK801-induced schizophrenia model.** MK801 disturbs N-methyl-D-aspartate receptor subtypes and their functions, which further results in a reduction of excitation of PV interneurons. Consequently, the “pathological” mEPSCs and mIPSCs imbalance of pyramidal cells and behavioral disorders occur. Sevoflurane, a potentiator of GABA_A_Rs, reverses the E/I imbalance and attenuates the abnormal behaviors. Abbreviations: E/I imbalance, excitation/inhibition imbalance; ErbB4, erb-b2 receptor tyrosine kinase 4; GAD67, glutamic acid decarboxylase67; GABA, γ-aminobutyric acid; GABA_A_R, GABA type A receptor; MK801, dizolcipine; mEPSCs, miniature excitatory postsynaptic currents; mIPSCs, miniature inhibitory postsynaptic currents; NMDAR, N-methyl-D-aspartate receptor; NRG1, Neuregulin 1; nAChRs, neuronal nicotinic acetylcholine receptors; PV, parvalbumin; PSD95, postsynaptic density protein 95.

The present study demonstrated that MK801 administration to neonatal mice induced a negative model of schizophrenia, including hypoactivity and less sociability. These abnormalities may be explained by changes to NMDAR subtypes and GABAergic neurotransmitter, and the consequential excitatory/inhibitory neurotransmission imbalance in the pyramidal neurons in laminar II-III of the PFC. All these changes can be reversed by sevoflurane administration (Fig. **6**). Furthermore, a low concentration of sevoflurane inhalation effectively reversed MK801-induced negative symptoms of schizophrenia. The good tolerance and effectiveness of sevoflurane together with dexmedetomidine in schizophrenia patients noted in the current study would encourage further a proper randomized controlled trial in comparison with sevoflurane and placebo treatment with a longer follow-up window (a few months) to evaluate the therapeutic effectiveness of sevoflurane while continuously monitoring undesired side effects in the long-term.

## Data Availability

The data that support the findings of this study is available from the corresponding authors on a reasonable request.

## LIST OF ABBREVIATIONS

BPRS-18: 18-item Brief Psychiatric Rating Scale
E/I: Excitation/Inhibition
GABA_A_Rs: γ-aminobutyric Acid Type A Receptors
GAD67: Glutamic Acid Decarboxylase67
MK801: Dizocilpine
mEPSCs: Miniature Excitatory Postsynaptic Currents
mIPSCs: Miniature Inhibitory Postsynaptic Currents
nAChRs: Neuronal Nicotinic Acetylcholine Receptors
NRG1: Neuregulin 1
NMDARs: N-methyl-D-aspartate receptors
NRG1: Neuregulin 1
OFT: Open Field Test
PANSS: Positive and Negative Syndrome Scale
PCP: Phencyclidine
PSD95: Postsynaptic Density Protein 95
PV: Parvalbumin
TEAEs: Treatment-emergent Adverse Events
α2A-AR: α_2A_-adrenoceptor
