# Supplementary material for: Sevoflurane Ameliorates Schizophrenia in a Mouse Model and Patients: A Pre-Clinical and Clinical Feasibility Study
Source: Curr Neuropharmacol. 2022 Nov 15;20(12):2369–80. doi: 10.2174/1570159X20666220310115846 (PMC9890293; doi:10.2174/1570159X20666220310115846)
Supplement: Supplementary file 1 [file CN-20-2369_SD1.pdf]

Supplementary Material

Sevoflurane Ameliorates Schizophrenia in a Mouse Model and Patients: A Pre-clinical and Clinical Feasibility Study

Tianyun Zhao[1,#], Ziwen Shi[1,#], Nongxi Ling[2,#], Jingwen Qin[1], Quancai Zhou[2], Lingzhi Wu[3], Yuansheng Wang[4], Chuansong Lin[2,*,$], Daqing Ma[3,*,$] and Xingrong Song[1,*,$]

[1]Department of Anesthesiology, Guangzhou Women and Children's Medical Center, Guangzhou Medical University, Guangzhou, China; [2]Department of Psychiatry, The Third People's Hospital of Xinhui District, Guangdong, China; [3]Division of Anaesthetics, Pain Medicine & Intensive Care, Department of Surgery & Cancer, Faculty of Medicine, Imperial College London, Chelsea and Westminster Hospital, London, UK; [4]Department of Anesthesiology, Jiangmen Central Hospital, Affiliated Jiangmen Hospital of Sun Yat-sen University, Guangdong, China

Supplementary Materials includes:

<u>Supplementary experimental methodology and trial protocol</u>

Materials and methods: file S1 of pre-clinical studies

Flowchart: File S2. Flowchart of clinical trial of patients' exclusion and enrolment

Clinical trial protocol: File S3

<u>Supplementary tables</u>

Table S1. Inclusion and Exclusion Criteria

Table S2. Clinical and Demographic Characteristics

Table S3. PANSS at Baseline, Week 1, Week 2, and the early response of week 1 and week 2 of each patient

<u>Supplementary Figures</u>

Fig. S1. Effect of MK801 administration during neonatal period on body weight

Fig. S2. Recording density maps showing social interaction defect

Fig. S3. Liver and kidney functions were not affected by the treatment compared to the baseline

Materials and methods: file S1 of pre-clinical studies

Subjects and Drugs

Subjects

Timed-pregnant Balb/c mice were purchased from Guangdong Medical Laboratory Animal Center, Guangzhou, China. They were housed in a temperature and humidity-controlled room (23 ± 1℃, 45-55%) of 12-hour light/dark cycle (lights on at 08:00 a.m.) with free access to water and food. The day of birth was designated as the postnatal day 0 (P0). On the P7, they (weighing 4-6 g) were randomly allocated into three groups: control group (CTRL group), MK801 group, and MK801+Sevoflurane group (MK801+SEV group). At the time of weaning (P21), mice were separated from their mothers and housed in group of four to six per cage.

Drugs administration

MK801 (Ref. M107, St. Louis, MO, USA) was used to induce negative symptoms of schizophrenia as reported previously [23]. It was dissolved in 0.9% sterile saline. At the P7, pups received intraperitoneal (i.p.) injection of 0.5 mg kg^{-1} MK801 in the MK801 group and MK801+SEV group or an equal volume of saline (CTRL group) twice a day for five consecutive days. Their body weights were measured every day during the treatment period and once/week since cessation of the treatments till the P31. During the P31-P35, the MK801+SEV group mice were placed in a 25 cm x 20 cm x 15 cm plexiglass chamber, and 1% sevoflurane was delivered via inlet of the chamber via a sevoflurane vaporizer (Easy fill/Cagemount, R58S, RWD Life Science Co.Ltd., Shenzhen, China) at 1 L min^{-1} in 30% oxygen balanced with nitrogen for 1 hr (10:00 am to 11 am) for five consecutive days. The concentration of sevoflurane was monitored with a gas monitor (BeneView T8, Mindray Bio-Medical Electronics Co.Ltd., Shenzhen, China) via the outlet of the chamber. The MK801 and CTRL groups received identical gases without sevoflurane under the identical setting for 1hr for 5 days. Open field test was performed at the P43-P45, and then three-chamber social test was conducted after two non-stimuli days at the P47. After behavioral tests (see below), they were sacrificed under terminated anesthesia and their brain samples were harvested for electrophysiological recordings, western blot and immunohistochemical analysis,

respectively. The experimental timeline is presented in Fig. 1A.

Behavioral tests

In order to avoid possible behavioral testing biases, mice were tested in a random order and all trails were carried out in the day at the same time period (9:00–16:00). In addition, the apparatus was cleaned with 70% ethanol after each trail to eliminate any effects from olfactory perception.

Open field test (OFT)

Open field test was adopted for assessing spontaneous locomotor activity. Mice were gently placed in the center of an open-top apparatus with 25 equal squares (50 cm × 50 cm × 40 cm) and allowed to move freely for 15 min. Distances and traces of 15 min movements were recorded with a video camera and analyzed using EthoVision XT 8.0 (Noldus, Wageningen, Netherlands), the total distance travelled and the number of squares crossed during recording period were calculated for further analysis.

Three-chamber sociability test

The three-chamber test was conducted to assess social interaction with time spent in a side chamber with a novel mouse in a wire cup versus time spent in a side chamber with an empty identical wire cup. A rectangular plexiglass box (60 cm X 40 cm X 20 cm) without a top cover was divided into three chambers (20 cm X 40 cm X 20 cm) with two partitions. Between each chamber, there is a 5-cm opening hole which can be closed or opened with a lever operated door. The middle chamber was empty, while the side chamber contained an empty wire cup or an identical wire cup with a novel sex- and weight-matched mouse in. The test was divided into two phases. In brief, after a ten-minute habituation period (phase 1), the test mouse was placed in the middle chamber and allowed free access to visit each chamber. For the social approach phase (phase 2), the mouse was placed in the central compartment and left to explore with either an empty black wire cup (in left side chamber) or a similar wire cup with a novel mouse inside (the rightside chamber). The apparatus and

wire cups were thoroughly cleaned with 70% ethanol between phases and after each trial. Behaviors were videotaped by an automated tracking software (TopScan/ObjectScan, Cleversystems, Leesburg, VA, USA). The time spent in each compartment, and the time explored (sniffing) within a 2 cm vicinity of the cup with a novel mouse in was calculated to assess sociability.

Western blot

PFC samples (n = 6 per group) were harvested for western blot analysis and dissociated in lysis buffer (containing protease inhibitors, 50 mM Tris–HCl, pH 7.6) on ice for 30 min and homogenized via ultrasonification (Ningbo scientz biotechnology CO. LTD, Ningbo, China). After centrifugation at 12,000 g for 10 min at 4°C, supernatants were collected and protein concentrations were measured with a BCA assay kit (Beyotime Institute of Biotechnology, China). Equivalent amounts of protein samples mixed with gel loading buffer (50 mM Tris-HCl, 10% SDS, 10% glycerol, 10% 2-mercaptoethanol, 2 mg ml^{-1} bromophenol blue) were boiled for 5 min and loaded to SDS-PAGE gels. The separated proteins were electrophoretically transferred to polyvinylidene difluoride membranes. Then the membranes were incubated in blocking buffer (5% fat-free milk in Tween20) for 2 hrs at room temperature and probed with primary antibodies: Rabbit anti-NR2A(1:1000, Cat.No.AB1555P, Millipore, USA), Rabbit anti-NR2B (1:1000, Cat.No.AB1557P, Millipore, USA), Rabbit anti-$GABA_A\alpha1$ (1:1000, Cat.No.224203, Synaptic Systems, Germany), Rabbit anti-$GABA_A\beta2$ (1:1000, Cat.No.224803, Synaptic Systems, Germany), Mouse anti-PSD95 (1:1000, Cat.No.MAB1596, Millipore, USA), Mouse anti-ErbB4 (1:1000, Cat.No.05-1133, Millipore, USA), Mouse anti-NRG1 (1:1000, Cat.No.sc-393006, Santa Cruz Biotechnology, USA), Rabbit anti-GAPDH (1:1000, Cat.No.5174, Cell Signaling Technology, USA) and Rabbit anti-β-Tubulin (1:1000, Cat.No.2128, Cell Signaling Technology, USA) overnight at 4°C. After washing in TBST for three times, the membranes were incubated with horseradish peroxidase (HRP) conjugated secondary antibodies diluted 1:5000 (Goat anti-Rabbit (1:5000, Cat.No.ab6721, Abcam, UK) or Rabbit anti-Mouse(1:5000, Cat.No.ab6728, Abcam, UK) for 2hrs at room temperature. The antigen-antibody complexes were detected by enhanced

chemiluminescence system (Bio-rad) and visualized by a computer image system (GENE GNOME Chemiluminescence apparatus, Quantity one, Bio-Rad Laboratories). Image processing and semi-quantification were performed with Image J software. Measurements were repeated independently at least 3 times for each experiment. Density of each band was normalized to the internal controls (β-tubulin or GAPDH).

Histology and immunohistochemistry

After antigen recovery, coronal paraffin sections (5 μm in thickness) were incubated with primary antibodies: mouse anti-parvalbumin (PV, 1:500, Millipore, Cat.No.MAB1572), or anti-GAD67 (1:1000, Millipore, Cat.No.MAB5406) at 4 °C. Signal was detected with Alexafluor 546-labeled fluorescent secondary antibodies (1:1000, Invitrogen, Carlsbad, CA, USA) for GAD67 positive cells, while signal for PV positive cells was detected with a mouse-rabbit ABC kit (PK-6200, Universal, Vector). Images were captured using camera system (SP8 and DMi8 DFC7000J, Leica, Germany). GAD67 and PV positive cells were calculated using Image J (NIH, USA) and expressed as cell numbers per mm^2.

Electrophysiological recording

Slice preparation

Coronal brain slices (250 μm) containing the PFC were cut with a vibratome (VT120S, Leica Microsystems) in ice cold solution containing (in mM): 75 sucrose, 85 NaCl, 2.5 KCl, 1.25 NaH_2PO_4, 4 $MgSO_4$, 0.5 $CaCl_2$, 24 $NaHCO_3$, 25 D-glucose saturated with 95% O_2/5% CO_2, and recovered in a chamber filled with artificial cerebrospinal fluid (ACSF) (in mM: 119 NaCl, 2.5 KCl, 1.25 NaH_2PO_4, 2 $MgCl_2$, 2 $CaCl_2$, 26 $NaHCO_3$, 10 D-glucose) saturated with 95% O_2 and 5% CO_2 at 32 ◦C before recording.

Whole-cell recording

After incubation, slices were transferred to a recording chamber where perfused at 2-3 ml min^{-1} with ACSF, saturated with 95% O_2 and 5% CO_2 and maintained at 32 ± 2 °C. The pyramidal neurons of PFC layer II/III were viewed with an Olympus microscope equipped with infrared DIC optics. The patch recording pipettes (4-6 MΩ) were filled with a solution (in

mM: 135 cesium methanesulfonate, 8 CsCl, 0.25 EGTA, 10 HEPES, 7 Na_2-phosphocreatine, 0.34 Na_3-GTP, 2.168 Mg-ATP, pH = 7.2-7.3, with CsOH) and access resistance of recorded cells was less than 30 MΩ. mEPSCs /mIPSCs were recorded at holding potential of -70 mV/0 mV in ACSF supplemented with 1 μM tetrodotoxin. Only cells with membrane potentials lesser than -65 mV and series resistance below 25 MΩ were included for further analysis. Cells were excluded if input resistance changed 20% over the entire experiment. Whole cell recordings were conducted by using Multiclamp 700B amplifier (Axon Instruments). All data were collected with 2 kHZ Bessel filter at a 10 kHz sampling frequency (DigiDATA 1550A, Axon Insteuments), and analyzed by Clampfit 10.0 software (Axon Instruments) following low-pass filtering at 1000 Hz. The synaptic response belonging to the inhibitory and excitatory amplitude was used to determine the E/I ratio (mEPSC amplitude/mIPSC amplitude) of the recorded pyramidal neuron.

Fig. S2. Flowchart showing patients' exclusion and enrolment

14 assessed for eligibility

4 patients were excluded:
1 refused to join in
1 immunologic disease
1 significant ECG abnormality
1 possibility of pregnancy

10 patients were included

9 patients completed all 6 trial treatments.
1 patient was stopped to participate because of postural hypotension after received one treatment.

Final analysis

Flowchart showing patients' exclusion and enrolment.

ECG: electrocardiograph.

Clinical Trial protocol: File S3

Section A

Efficacy and safety of adjunctive low-concentration sevoflurane in the treatment of hospitalized patients with acute schizophrenia: An open-labelled single arm trial

Study Drug: Sevoflurane

Principal Investigator: Xingrong Song, MD

Study Design: Xingrong Song, MD, Daqing Ma, MD, PhD, FRCA, and Chuansong Lin, MB

Name of Institute: Department of Anesthesiology (Xingrong Song, MD), Guangzhou Women and Children's Medical Center, Guangzhou Medical University, Guangzhou, 510623, China; Division of Anaesthetics, Pain Management and Intensive Care (Daqing Ma, MD, PhD, FRCA), Department of Surgery and Cancer, Imperial College London, Chelsea and Westminster Hospital, London, UK, and Department of Psychiatry (Chuansong Lin, MB), The Third People's Hospital of Xinhui District, Guangdong, 529100, China.

Version of Protocol: Second version (20190716)

Contents

1. Background

2. Purpose of the study

3. Recruitment of subjects

4. Study design

5. Intervention protocol

6. Data collection

7. Outcomes

8. Adverse events

9. Severe adverse events

10. Data management

11. Statistical analysis

12. Quality control and quality assurance

13. Ethics requirement and written informed consent

14. Preservation of documents

15. Declaration of interests

Background

Schizophrenia is a chronic and devastating psychiatric disorder that affects approximately 1% of population [1]. Despite recent pharmacological advances, current antipsychotic medications have limited effectiveness on negative symptoms, in addition to a wide range of side effects including cognitive impairment, weight gain, motor impairments, metabolic disturbances, prolactin abnormalities and cardiovascular risks [50]. Although etiology and mechanisms of schizophrenia remain elusive, it has been considered that GABAergic deficits, and consequently disrupted excitatory and inhibitory (E/I) balance in the prefrontal cortex, serve central roles in the pathophysiology of schizophrenia [12]. Indeed, previous studies suggested changes to various aspects of the inhibitory system in schizophrenia, including decreases of the interneuronal marker expression and interneuronal numbers, diminished GABA synthesis, multiple GABA gene alterations, altered GABA subtype receptors in cortex and hippocampus [12]. Thus, targeting GABAergic neurons and their associates may shed light to a novel therapeutic development for psychotic disorders. There are no effective drugs targeting the GABAergic system currently available in clinical practice, although significant research effort is in place to explore the feasibility of targeting GABA system in schizophrenia [11]. In addition, a general consensus is that any medications for schizophrenia treatment should have the properties of rapid onset of action, calmness without sedation, easy administration, non-invasive, non-traumatic/non-coercive, safety profile and favorable tolerability [51].

Sevoflurane, a commonly used volatile anesthetic for surgical anesthesia, is a potentiator of γ-aminobutyric acid type A ($GABA_A$) receptors and can induce a rapidly and well-controlled

sedation [14]. Because of these advantages, sevoflurane and other inhalational anesthetics are increasingly used as general sedatives for invasive diagnostic and therapeutic procedures, mechanical ventilation and management of agitation in intensive care unit. The concentration required for sedation is approximately one third (0.3 minimum alveolar concentration) of the concentration required for general anesthesia with less incidence of nausea and vomiting [14]. All these led us to explore the therapeutic value of sevoflurane to ameliorate schizophrenia symptoms. In this proof-of-concept study, we investigated the effectiveness and safety of sub-anesthetic, sedative dose of sevoflurane (0.3-0.5 MAC) as an adjuvant therapy to schizophrenia patients undering routine antipsychotic treatments.

2. Purpose of the study

To investigate the effectiveness and safety of low-concentration sevoflurane (1%) in schizophrenia patients experiencing an acute exacerbation of psychosis.

3. Recruitment of subjects

Potential participants will be screened from newly admitted patients by investigators.

3.1 Inclusion criteria

3.1.1 Subjects meeting the DSM-V, ICD-10 criteria for schizophrenia;

3.1.2 Male or female Chinese subjects age of 18-65 years;

3.1.3 Subjects are in-hospital patients;

3.1.4 Subjects are in the phrase of acute schizophrenia with a score from 70 to 120 of the PANSS scale and are appropriate to receive sevoflurane inhalation according to the investigators' judgment;

3.1.5 Subjects with a minimum score of 14 of the PANSS-excited components (PANSS-EC) score with at least 1 item no less than 4. (P4. excited, P7. Hostility, G4. Physical tension, G8.

Uncooperative, G14. Impulse control disorder);

3.1.6 Evidence of a subject (or a legal representative) signed and dated on written informed consent document.

3.2 Exclusion criteria

Patients will be excluded if they meet any of the following criteria:

3.2.1 Acute episode due to delirium, seizures, affective psychosis, poisoning, or substance abuse withdrawal reactions;

3.2.2 Relevant history or current presence of any cardiovascular, respiratory, neurological, renal, hepatic, endocrine, immunologic or other systemic disease;

3.2.3 Confirmed clinically significant abnormal laboratory measurements; Clinically significant ECG abnormality;

3.2.4 Subjects with a history of QTc prolongation or a pre-drug QTc of 450 msec or greater; Subjects with serum K+ or Mg2+ beyond the normal range;

3.2.5 A history of malignant syndrome or tardive dyskinesia history; Pregnant or lactating women;

3.2.6 Monoamine oxidase antidepressants were administered within 2 weeks and long-acting antipsychotics were administered within 1 month;

3.2.7 Participation in other studies.

3.3 Criteria of drop out

3.3.1 Participants and/or their legal representatives withdrawn consents;

3.3.2 Loss to follow-up;

3.3.3 Exit decision made by the investigators or attending physicians (poor compliance, occurrence of severe complications or severe adverse events);

For drop out cases, the detailed reasons will be recorded and the primary therapeutic effects recorded in the last time will be regarded as the final results. The Case Report Forms (CRFs) of these cases will be preserved for future reference.

3.4 Criteria of Rejection

Enrolled cases who meet any of the following criteria will be excluded from further data analyses.

3.4.1 Study drug is not administered;

3.4.2 No study record;

3.4.3 Unable to evaluate the effectiveness and safety because of the use of any prohibited drugs.

For rejected cases, the detailed reasons will be recorded and CRFs will be preserved for reference. The results of these cases will be excluded for further analysis of therapeutic effects.

3.5 Criteria of Study Interruption

Study will be interrupted in the following conditions:

3.5.1 Severe safety problem occurred during the study;

3.5.2 Serious mistake found in the protocol;

3.5.3 Fund or management problem of the investigators;

3.5.4 Study cancelled by administrative authority.

Study interruption may transient or permanent. All recorded CRFs will be preserved for reference in case of study interruption.

4. Study design

4.1 Type of the study

The study is a single-center, open-labelled, single arm trial.

4.2 Sample size calculation

Simon's two stage design was used in this proof of concept study. Based on findings of an earlier study, the percentage of patients with at least 30% improvement in total PANSS at 2 weeks was about 30% (*14*). In this study, a 70% or higher early response rate was set as the target of "good" and 30% was set as "poor". The optimal two-stage design to test the null

hypothesis that P<=0.300 versus the alternative that P>=0.700 has an expected sample size of 6.08. After testing the drug on 2 patients in the first stage, the trial was planned to be terminated if 0 respond. If the trial goes on to the second stage, a total of 10 patients was planned to be studied. If the total number of patients responding is less than or equal to 5, the drug was rejected.

5. Intervention protocol

5.1 Intervention drug

5.1.1 Name: Sevoflurane for inhalation (Baxter, Illinois, USA).

5.1.2 Product specification: 250 ml.

5.2 Intervention procedure

Patients receive routine antipsychotic medications (based on established clinical guidelines) including one or two antipsychotics limited to risperidone, paliperidone, aripiprazole, and olanzapine. After obtained written informed consent, patients will be arranged to fast for 8 hrs first and then receive sevoflurane treatment. Two fully trained anesthetists to administrate sevoflurane inhalation. Intravenous access will be established and lactated Ringer's solution or 0.9% saline will be infused at the rate of 80-120 ml h^{-1} according to their body weight. Participants will be monitored with electrocardiography, pulse oximetry, non-invasive blood pressure, bispectral index (BIS), end-tidal partial pressure of carbon dioxide, and temperature. Dexmedetomidine (Hengrui Pharmaceutical Co. LTD, Jiangsu, China) with a loading dose of 0.5 μg kg^{-1} will be intravenously administrated by a pump for 15 min and then maintained with a rate of 0.1-0.3 μg $kg^{-1}.h^{-1}$ before sevoflurane administration. Sevoflurane (Baxter, Illinois, USA) will be delivered in 2.5 L min^{-1} oxygen enriched air (50% oxygen and 50% air) for 5 hrs using a face mask under spontaneous respiration. Sevoflurane will be given at 4.0% for first 5-min duration and then gradually decreased to 1.0 % and adjusted between 0.5-1.2% according to the level of sedation. During the sedation, BIS will be maintained between 50-65, and the BARS (Behavioral Activity Rating Scale, BARS) will be assessed every 15 min to maintain 2-3. After

discontinuing the sevoflurane inhalation, patients will be continued to monitor until they gain full consciousness. The same treatment will be repeated for 6 times with intervals of 1-2 days for a total of two weeks.

6. Data collection

6.1 Basic data

6.1.1 Demographic data, medical history, medication history, diagnosis, length of the illness;

6.1.2 Electrocardiogram, Hematological and biochemical test results;

6.1.3 PANSS total score.

6.2 Data during the sevoflurane inhalation treatment

6.2.1 type and dose of antipsychotics;

6.2.2 Concentration of sevoflurane, bispectral index (BIS), minimum alveolar concentration (MAC), pulse oximetry, non-invasive blood pressure, heart rate, end-tidal partial pressure of carbon dioxide, and temperature, BARS (Behavioral Activity Rating Scale, BARS).

6.3 *Data after the sevoflurane inhalation treatment*

6.3.1 the Positive and Negative Syndrome Scale (PANSS) total score;

6.3.2 the 18-item brief Psychiatric Rating Scale (BPRS-18);

6.3.3 Occurrence of adverse events during the 2-week treatment. If an adverse event occurs, it will be followed up and remedy until its disappearance or therapy ends;

6.3.4 Type and dose of antipsychotics during the treatment;

6.3.5 Use of benzodiazepines, haloperidol, electroconvulsive therapy, and other remedies during the treatment.

7. Outcome

7.1 Primary outcome

The percentage of the early response at week 2, and the clinical response was defined as a minimum 30% reduction in Positive and Negative Syndrome Scale (PANSS) total score.

7.2 Secondary outcomes

The secondary endpoints were the change of Brief Psychiatric Rating Scale (BPRS-18) from the baseline to week 1 and week 2, and the early response rate at week 1.

8. Adverse events

8.1 Definition

An adverse event indicates any unpredictable, unfavorable medical event that is associated with any medical intervention and occurs during the study period. It can be related to the study drug administration or otherwise. It can manifest as any uncomfortable signs (including abnormal laboratory findings), symptoms or transient morbidity.

8.1.1 Predicted adverse events in this study:

8.1.1.1 Bradycardia: heart rate < 55 beats per minute or, in case of a baseline value < 69 beats per minute, a decrease of more than 20% from baseline (before study drug infusion);

8.1.1.2 Hypotension: systolic blood pressure < 95 mmHg or, in case of a baseline value < 119 mmHg, a decrease of more than 20% from baseline;

8.1.1.3 Tachycardia: heart rate > 100 beats per minute or, in case of a baseline value > 83 beats per minute, an increase of more than 20% from baseline;

8.1.1.4 Nausea and vomiting.

8.2 Management

8.2.1 Therapy will be provided according to routine clinical practice;

8.2.2 Management of predicted adverse events in this study:

8.2.2.1 Bradycardia: administration of medication (atropine iv bolus and/or isoprenaline iv infusion only) and/or adjustment of study drug infusion;

8.2.2.2 Hypotension: intravenous fluid bolus, administration of medication (ephedrine/ phenylephrine iv bolus, dopamine/norepinephrine iv infusion), and/or adjustment of study drug infusion;

8.2.2.3 Tachycardia: administration of medication (esmolol/diltiazem iv infusion) and/or adjustment of study drug infusion;

8.2.2.4 Nausea and vomiting: dexamethasone/5-HT3 receptor antagonist (iv infusion).

8.3 Record

8.3.1 Any adverse events will be documented, including occurrence, type, time of diagnosis, management, duration of persistence, and sequelae;

8.3.2 Any adverse event will be followed up until it is completely resolved or therapy termination.

9. Severe adverse events

9.1 Definition

A severe adverse event indicates any unpredictable medical events that lead to death, threat of life, prolonged length of hospital stay, persistent disability or dysfunction, or other severe event.

9.2 Management

In case of any severe adverse events, the sevoflurane inhalation therapy will be stopped and treatment will be initiated immediately.

9.3 Record and report

9.3.1 In case of any severe adverse event, apart from active treatment and record as above, the principal investigator and the Ethics Committee will be informed within 24 hrs in written report;

9.3.2 In case of study drug related death, immediately stop the clinical trial, report the event to the Ethics Committee as soon as possible, record in detail and carefully preserve the related documents;

9.3.3 Any severe adverse event must be followed up until it is completely resolved or when therapy is ended.

10. Data management

10.1 Investigators should promptly, completely, and correctly record data in the CRF according to original observation;

10.2 Supervisors will monitor whether the study is carried out according to the protocol. The completed CRFs, after signed by the supervisor, will be sent to an investigator who is responsible for data management;

10.3 Data input will be performed by one investigator and checked out by another independent researcher. CRFs will be stored safely;

10.4 Data management will be inspected by the Clinical Research Ethics Committee of Guangzhou women and children's Hospital.

11 Statistical analysis

11.1 General principles

The efficacy and safety analysis were conducted in the full analysis set, which included all patients who received at least one treatment of sevoflurane inhalation and had at least one evaluation post-baseline. The early response of BPRS-18 total score at week 1 and week 2 was reported as mean with standard deviation.

11.2 Patient recruitment and drop-out status

The status of patient recruitment and drop-out will be summarized and listed.

11.3 Demographics and baseline characteristics

Demographic information and baseline characteristics will be presented.

12 Quality control and quality assurance

12.1 For investigators/care givers

12.1.1 Trial protocol will be thoroughly explained to all investigators/care givers before the start of the trial. The trial protocol must be strictly adhered throughout the trial period;

12.1.2 All expected and unexpected findings will be documented promptly and correctly in order to guarantee the reliability of the values;

12.1.3 The monitors and instruments that are used during the study period will be checked and corrected regularly in order to guarantee their normal work;

12.1.4 Data analysis will be performed by the biostatisticians and investigators;

12.1.5 Any conclusions must be derived from the original data.

12.2 For participants

12.2.1 Possible benefits and risks associated with the study drug administration will be clearly explained to every potential participant;

12.2.2 Written informed consent must be signed by every enrolled patient, or by the authorized representatives of the patient;

12.3 Study termination

12.3.1 If a study drug-related death occurs during the study period, the study will be stopped.

A report will be sent to the Ethics Committee. Restart of the study will need an approval from the Ethics Committee;

12.3.2 The study will be terminated after accomplishment of patient recruitment and data collection. Decision will be made by the principal investigator.

13 Ethics requirement and written informed consent

13.1 Helsinki declaration and Chinese guidelines of Good Clinical Practice will be strictly followed. The study protocol must be approved by the Ethics Committee before the study can be started;

13.2 For every potential participant, investigators have the responsibilities to fully explain the study purpose, procedures, as well as possible risks in a written informed manner. They must let every potential participant know that he/she has the right to withdraw his/her

consent at anytime during the study period. Every potential participant must be given a written informed consent. Every participant or the authorized surrogate of the patient must sign the consent before they can be enrolled in the study. Written informed consents will be kept as a part of the clinical trial documents.

14 Preservation of documents

Investigators will carefully preserve all documents and data of the clinical trial according to the Good Clinical Practice requirement.

15 Declaration of interests

The authors report no conflicts of interest in this work.

Section B

Efficacy and safety of adjunctive low-concentration sevoflurane in the treatment of hospitalized Chinese patients with acute schizophrenia: An open-labelled single arm trial

Description of the trial and written informed consent

1. We invite you to participate in this study

We invite you to participate in this open-labelled single arm study investigating the " Efficacy and safety of adjunctive low-concentration sevoflurane in the treatment of hospitalized Chinese patients with acute schizophrenia”. The study is organized by Guangzhou Women and Children's Medical Center and will be performed in the Third People's Hospital of Xinhui District, Guangdong. We are expected to enroll 20 participants among inpatients with schizophrenia who are experiencing an acute exacerbation episode.

Before you decide to participate in the study, please read the following description carefully which will help you to understand the purpose and contents of the study, to understand the potential benefits from the study that it may bring to you, as well as the potential risks that you may encounter during the study. You are welcome to discuss your concerns with your doctors, relatives and/or friends freely before making the decision. If you have any questions or would like to get more information about the trial, please do contact us.

2. What is the purpose of this study?

Schizophrenia is a chronic, devastating, recurrent lifelong psychiatric disorder, which affects approximately 1% of the world population and causes globally socio-economical and healthcare problem in our societies. At present, the pathogenesis of schizophrenia is complex, and the exact mechanism has not been established. Antipsychotics are the standard medication for schizophrenia and, indeed, dopamine or/and serotonin modulating antipsychotics remain the primary approved treatment for schizophrenia.

In recent years, some researchers have proposed the glutamate and γ-aminobutyric acid hypothesis on the pathogenesis of schizophrenia. But currently there are no relevant clinical

drugs available. Sevoflurane, a commonly used volatile anesthetic for anesthesia, is a potentiator of γ-aminobutyric acid type A ($GABA_A$) receptors and can induce a rapidly and well-controlled sedation through non-invasive face mask with minimum side effects. Studies have shown that sevoflurane at low concentrations has neuroprotective effects and can improve cognitive function. In addition, we found that the behavioral, electrophysiological and related protein expression in the cortex were reversed after low-concentration sevoflurane treatment in the mouse model of schizophrenia. Therefore, we believe that sevoflurane can be used for the treatment of schizophrenia through GABAergic receptors. In particular, can rapidly control the symptoms of acute episode and avoid the risk of escalation. You will be treated with low-concentrations of sevoflurane for 2 weeks. The purpose of this study is to provides new directions for studying the clinical treatment of schizophrenia and drugs research.

3. Do I have to participate in?

The decision to participate in the study is entirely voluntary. You make the decision to take part in or not, and can withdraw consent at any time without giving any reasons during the study. If you decide to participate, please sign the written informed consent form. You can keep a signed informed consent form and a copy of study description. Withdrawal from the study does not affect your treatments throughout your hospital stay. In such case, your data and information will not be used in the study. On the other hand, if you or we think the study is affecting your normal treatment or outcome, the researchers will also stop the study.

During the period of study participation, please tell researchers your true medical history and current physical status, as well as whether you have participated in any other studies currently or recently, or if you have any newly developed discomfort. If you do not abide the study protocol or develop any study-related harm, the researchers can terminate your participation in the study.

4. If I participate, what do I need to do?

If you agree to participate in this study, we will give a recruitment number and establish medical records for you. After admission, we will collect your demographic and baseline information and routinely perform the assessment using the Positive and Negative Symptom Scale (PANSS) and the Brief Psychiatric Rating Scale, which will take up 30 minutes of your time. We will evaluate your condition weekly for the first two weeks of treatment, and monitor the possible side effects and treatment-related complications. After discharge from the hospital, we will call you to assess your maintenance therapy, your health status and record the occurrence of other complications. All the above inspection and assessment are free of charge and the only thing you need to do is to cooperate with our investigators.

5. Will participation in this study bring me extra cost or reward?

This research is supported by special research fund, and participation in the study will not bring you extra cost or gain you any extra rewards.

6. Do I get any benefit from participating in the study?

This study will help you to relieve your acute episode symptoms as early as possible, such as positive symptoms, negative symptoms, agitation, depression, anxiety, and cognitive function decline. It will be beneficial to reduce the adverse effects caused by antipsychotics, achieve optimal prognosis, and prepare for returning to the community and restoration of social function. In addition, the findings of this study may change or guide the future antipsychotic discovery work to invent more efficacy, and rapid onset drug with less side effects which will bring benefit to more patients.

7. What kind of treatment will I receive in this study?

Conventional antipsychotics regimen will not be changed no matter whether you participate in this study or not during your hospitalization. Since it is a single arm, open-labelled clinical trial, you will have the only choice to be enrolled in the experimental group to receive low-concentration sevoflurane inhalation treatment when you decide to be a participant. Participants will receive low-concentration sevoflurane (0.3-0.5 MAC) inhalation for 5 hrs

using a face mask under spontaneously respiratory. During the sedation, BIS was maintained between 50-65, and the BARS (Behavioral Activity Rating Scale, BARS) was assessed every 30 min to maintain 2-3. The same treatment was repeated for 6 times with intervals of 1-2 days for a total of two weeks.

8. Are there any potential risks or adverse effects to me?

Commonly encountered adverse events (incidence > 1%): nausea, vomiting, hypotension and cough, excitement, drowsiness, chills, bradycardia, dizziness, increased salivation, respiratory disorders, hypertension, tachycardia, laryngeal spasm, fever, headache, hypothermia, increased SGOT (Serum glutamic-oxaloacetic transaminase (synonymous with AST)).

Accidental adverse reactions to sevoflurane (incidence < 1%): Arrhythmia, increased LDH, increased SGPT (Serum glutamic-pyruvic transaminase (synonymous with ALT)), hypoxemia, transient apnea, leukocytosis, supraventricular contraction, asthma, increased creatinine, urinary retention, glycosuria.

Severe adverse reactions: malignant hyperthermia and acute renal failure were rarely reported.

Any adverse events occurred during the study period will be managed promptly according to routinely clinical practice. In case of any harmful consequence resulted directly from the study, participant will be compensated according to the corresponding legal provisions.

9. Will my personal information be confidential?

Some data obtained from you during the study will be published in the form of scientific papers, but your personal information (including name, age, education and etc.) will be kept confidential and your personal privacy will be protected according to law. Non-research team personnel will not be allowed to obtain your information, unless permitted by yourself. Your information (recorded in written or other forms) will be preserved for 5 years and then destroyed 5 years after the end of the study. If the information needs to be preserved for more than 5 years, we will obtain your permission by telephone, and will inform you how

long and in what way your information will be preserved and used in the future. All research members and institutions involved in the study will maintain confidentiality. In order to ensure that the study is carried out in accordance with the regulations, administrative members or the ethics committee members may access your personal information when necessary.

10. How can I get more information?

If you have any questions about the study, or suffer from any discomfort and injury during the study, or want to obtain more information of the study, please do not hesitate to contact the research members: Xingrong Song, MD and Tianyun Zhao, MD. Tel: 020 38076457.

If you have any questions regarding the ethical issues of the study, please contact the Clinical Research Ethics Committee of Guangzhou women and children's medical center. Tel: 020-38367270.

When necessary, you can also contact the Department of Medical Service of Guangzhou women and children's medical center. Tel: 020-38367273.

“Efficacy and safety of adjunctive low-concentration sevoflurane in the treatment of hospitalized Chinese patients with acute schizophrenia: An open-labelled single arm trial”

Signature page

Informed consent and signature:

I have read the informed consent for this trial carefully. The study protocol has been explained to me in full detail. I totally understand the purpose and nature of the study, as well as my rights and risks during the study.

I would like to participate in the study voluntarily; and I can confirm that I cooperate with the research members according to the study protocol and the contents listed in informed consent and participate in the trial throughout the entire course of the study.

Patient

(or legally authorized representative)

________________________________ ____________ ______

Signature Date

Printed Name

(or legally authorized representative)

____

<Relationship between legally authorized representative and patient (e.g., parent or legal guardian)>

Person Obtaining Consent

____ ________________ ______

Printed Name & Title Signature Date

Section C

Positive and Negative Syndrome Scale (PANSS) items for inclusion-score sheet

Please enter the score for the term which best describes the patient's condition.

1 = absent, 2 = minimal, 3 = mild, 4 = moderate, 5 = moderate severe, 6 = severe,7 = extreme

Positive Scale

1	Delusions	
2	Conceptual Disorganization	
3	Hallucinatory behavior	
4	Excitement	
5	Grandiosity	
6	Suspiciousness/persecution	
7	Hostility	

Negative Scale

1	Blunted affect	_______
2	Emotional withdrawal	_______
3	Poor rapport	_______
4	Passive/apathetic and social withdrawal	_______
5	Difficulty in abstract thinking	_______
6	Lack of spontaneity and flow of conversation	_______
7	Stereotyped thinking	_______

General Psychopathology Scale

1	Somatic concern	_______
2	Anxiety	_______
3	Guilt feelings	_______
4	Tension	_______
5	Mannerisms and posturing	_______
6	Depression	_______
7	Motor retardation	_______
8	Uncooperativeness	_______
9	Unusual thought content	_______
10	Disorientation	_______

11	Poor attention	_______
12	Lack of judgment and insight	_______
13	Disturbance of volition	_______
14	Poor impulse control	_______
15	Preoccupation	_______
16	Active social avoidance	_______

Total PANSS Score: _____________

18-item Brief Psychiatric Rating Scale (BPRS-18)

Please enter the score for the term which best describes the patient's condition.

0 = not assessed, 1 = not present, 2 = very mild, 3 = mild, 4 = moderate, 5 = moderate severe,6 = severe, 7 = extreme severe

1	Somatic concern	
2	Anxiety	
3	Emotional withdrawal	
4	Conceptual disorganization	
5	Guilt feelings	
6	Tension	
7	Mannerisms and posturing	
8	Grandiosity	
9	Depressive mood	
10	Hostility	
11	suspiciousness	
12	hallucinatory behavior	
13	Motor retardation	
14	uncooperativeness	
15	Unusual thought content	
16	blunted affect	
17	excitement	
18	disorientation	

Behavioral Activity rating Scale (BARS)

1	difficult or unable to rouse
2	asleep, but responds normally to verbal or physical contact
3	drowsy, appears sedated
4	quiet and awake (normal level of activity)
5	signs of overt (physical or verbal) activity, calms down with instruction
6	extremely or continuously active, not requiring restraint
7	violent, requires restraint

Table S1. Inclusion and Exclusion Criteria

Inclusion Criteria	Exclusion Criteria
1. Subjects meeting the DSM-V, ICD-10 criteria for schizophrenia	1. Acute episode due to delirium, seizures, affective psychosis, poisoning, or substance abuse withdrawal reactions
2. Male or female Chinese subjects age of 18-65 years	2. Relevant history or current presence of any cardiovascular, respiratory, neurological, renal, hepatic, endocrine, immunologic or other systemic disease
3. Subjects are in-hospital patients at screening phase, and can remain in hospital during study period	3. Confirmed clinically significant abnormal laboratory measurements; Clinically significant ECG abnormality
4. Subjects are in the phase of acute schizophrenia with a score from 70 to 120 of the PANSS scale and are appropriate to receive sevoflurane inhalation according to the investigators' judgment	4. Subjects with a history of QTc prolongation or a pre-drug QTc of 450 msec or greater; Subjects with serum K+ or Mg2+ beyond the normal range
5. Subjects with a minimum score of 14 of the PANSS-excited components (PANSS-EC) score with at least 1 item no less than 4. (P4. excited, P7. Hostility, G4. Physical tension, G8. Uncooperative, G14. Impulse control disorder)	5. A history of malignant syndrome or tardive dyskinesia history; Pregnant or lactating women
6. Evidence of a subject (or a legal	6. Monoamine oxidase antidepressants

representative) signed and dated on written informed consent document	were administered within 2 weeks and long-acting antipsychotics were administered within 1 month
	7. Participation in other studies

Table S2. Clinical and Demographic Characteristics

Characteristics	
Age (years), (Mean ± SD)	34.3 ± 9.73
Gender, No. (%)	
Male	7 (70%)
Female	3 (30%)
Race (Han nationality), n (%)	10 (100%)
Length of illness (years), (Mean ± SD)	10.4 ± 8.62
Length of illness (years), range	1-31
Marital status (Married), No. (%)	3 (30%)
Body weight (Kg), (Mean ± SD)	59.20 ± 11.12
Baseline PANSS total scores, (Mean ± SD)	93.60 ± 15.81
Baseline BPR-S scores, (Mean ± SD)	52.80 ± 9.26

Abbreviations: BPR-S, Brief Psychiatric Rating Scale; PANSS, Positive and Negative Syndrome scale; SD, standard deviation.

Table S3. PANSS at Baseline, Week 1, Week 2, and the early response of week 1 and week 2 of each patient

Patients	Baseline	Week 1	Week 2	Reponse-1 (%)	Response-2 (%)
1	88	52	49	62.07	67.24
2	75	54	45	46.67	66.67
3	91	82	57	14.75	55.74
4	95	93	77	3.08	27.69
5	120	108	98	13.33	24.44
6	104	57	54	63.51	67.57
7	118	95	77	26.14	46.59
8	83	55	48	52.83	66.04
9	85	53	44	58.18	74.55
10	77	44	43	70.21	72.34

Abbreviations: PANSS, Positive and Negative Syndrome scale.

Supplementary Fig. S1

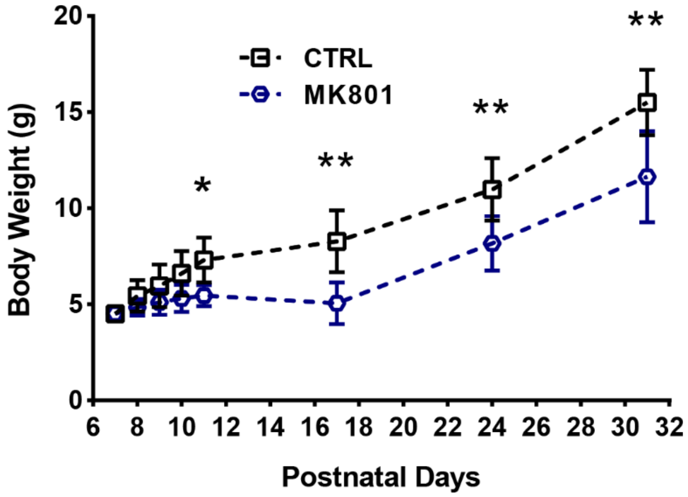

Fig. S1. Effect of MK801 administration during neonatal period on body weight

The body weight of the MK801 group was lower after 5-day systemic administration of MK801 (0.5 mg kg^{-1}, i.p., twice a day) compared with CTRL group, and this difference persisted thereafter. Data are analysed by two-way repeated measures ANOVA followed by Bonferroni's *post-hoc* test. Data are represented as mean ± SD (n = 18); **P* < 0.05; ** *P* < 0.01.

Supplementary Fig. S2

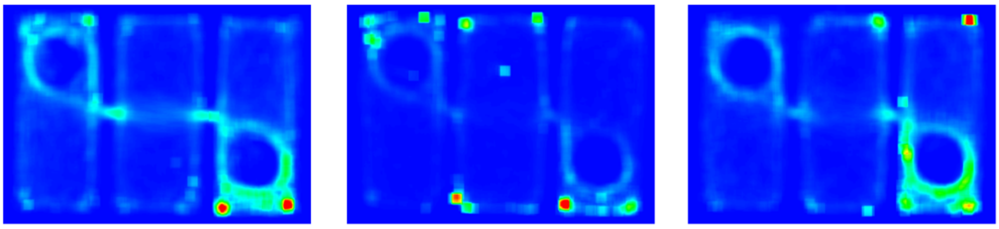

Fig. S2. Examples of recording density maps from CTRL (left), MK801 (middle), and MK801+SEV cohort (right).

Supplementary Fig. S3

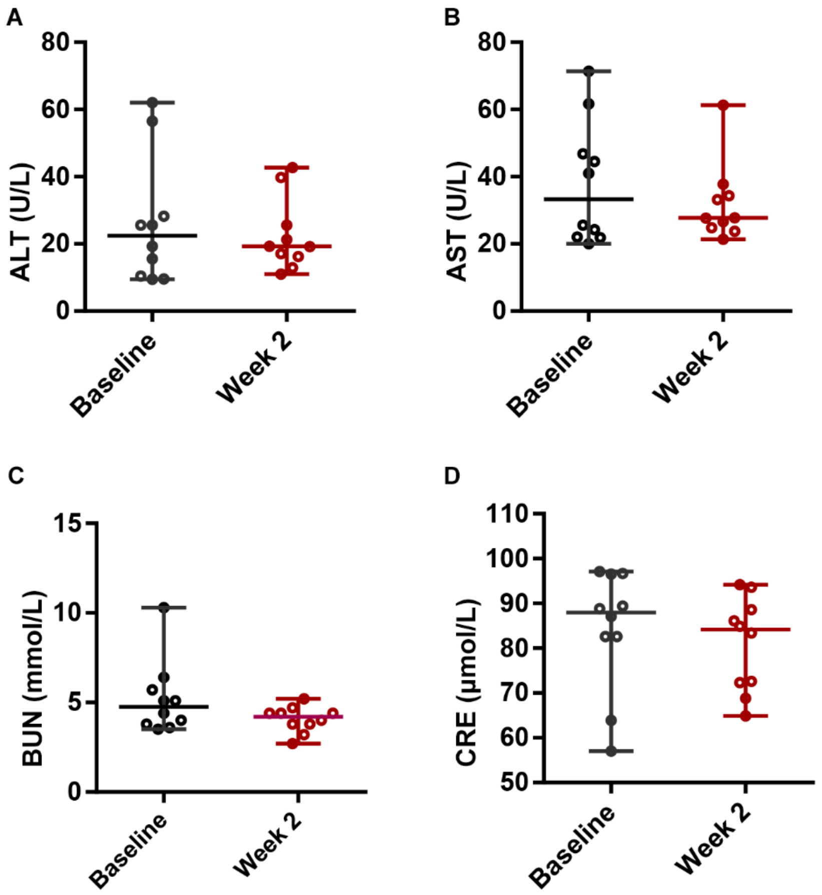

Fig. S3. Liver and kidney functions were not affected by the treatment when compared to the baseline. Data are represented as median with range (n = 10). Abbreviations: ALT, Alanine aminotransferase; AST, aspartate aminotransferase; BUN, urea nitrogen; CRE: Creatinine.
